# Improving quantification accuracy of a nuclear Overhauser enhancement signal at −1.6 ppm at 4.7 T using a machine learning approach

**DOI:** 10.1088/1361-6560/ada716

**Published:** 2025-01-17

**Authors:** Leqi Yin, Malvika Viswanathan, Yashwant Kurmi, Zhongliang Zu

**Affiliations:** 1 Vanderbilt University Institute of Imaging Science, Vanderbilt University Medical Center, Nashville, TN, United States of America; 2 School of Engineering, Vanderbilt University, Nashville, TN, United States of America; 3 Department of Biomedical Engineering, Vanderbilt University, Nashville, TN, United States of America; 4 Department of Radiology and Radiological Sciences, Vanderbilt University Medical Center, Nashville, TN, United States of America

**Keywords:** chemical exchange saturation transfer (CEST), nuclear Overhauser enhancement (NOE), machine learning, tumor

## Abstract

*Objective.* A new nuclear Overhauser enhancement (NOE)-mediated saturation transfer MRI signal at −1.6 ppm, potentially from choline phospholipids and termed NOE(−1.6), has been reported in biological tissues at high magnetic fields. This signal shows promise for detecting brain tumors and strokes. However, its proximity to the water peak and low signal-to-noise ratio makes accurate quantification challenging, especially at low fields, due to the difficulty in separating it from direct water saturation and other confounding signals. This study proposes using a machine learning (ML) method to address this challenge. *Approach.* The ML model was trained on a partially synthetic chemical exchange saturation transfer dataset with a curriculum learning denoising approach. The accuracy of our method in quantifying NOE(−1.6) was validated using tissue-mimicking data from Bloch simulations providing ground truth, with subsequent application to an animal tumor model at 4.7 T. The predictions from the proposed ML method were compared with outcomes from traditional Lorentzian fit and ML models trained on other data types, including measured and fully simulated data. *Main results.* Our tissue-mimicking validation suggests that our method offers superior accuracy compared to all other methods. The results from animal experiments show that our method, despite variations in training data size or simulation models, produces predictions within a narrower range than the ML method trained on other data types. *Significance.* The ML method proposed in this work significantly enhances the accuracy and robustness of quantifying NOE(−1.6), thereby expanding the potential for applications of this novel molecular imaging mechanism in low-field environments.

## 1. Introduction

Chemical exchange saturation transfer (CEST) is a rising molecular imaging mechanism in MRI, relying on the saturation transfer effect between water protons and solute protons that are either exchangeable or have coupling (Ward *et al* 2000, Zhou and van Zijl 2006, van Zijl and Yadav 2011, Liu *et al* 2013, Kim *et al* 2015, Wu *et al* 2016, van Zijl *et al* 2018, Vinogradov 2019). Nuclear Overhauser enhancement (NOE)-mediated saturation transfer is a variation of the CEST effect, relying on the dipolar interaction between water protons and solute protons that are restricted in their motion (Anderson and Freeman 1962, Vogeli 2014, Solomon 1955). During CEST/NOE imaging, a radio frequency (RF) saturation block selectively saturates solute protons, which then interact or exchange with water protons. After an extended saturation period, a cumulative effect of this exchanging or coupling induces a substantial attenuation in the water signal. By measuring the change in water signal, CEST/NOE can provide an amplification process to detect solute molecules with low concentrations or extremely short transverse relaxation time (*T*_2s_) that would otherwise remain unobservable (Wolff and Balaban 1990). Traditionally, a CEST Z-spectrum, which plots the CEST signal against the frequency offset of the saturation pulse, is measured. This allows for the observation of multiple CEST/NOE effects, as indicated by the dips on the Z-spectra.

In biological tissues, two main NOE effects have been observed at around −3.5 ppm and −1.6 ppm, referred to as NOE(−3.5) and NOE(−1.6), respectively. The NOE(−3.5) signal, which originates from mobile macromolecules, including proteins and membrane lipids (van Zijl *et al* 2003, Goerke *et al* 2017, Zhao *et al* 2023a), has been extensively studied. Its dip can be observed at various magnetic fields from 3 T to 15.2 T (Jones *et al* 2012, 2013, Zaiss *et al* 2015a, Xu *et al* 2016, Heo *et al* 2016b, 2016c, Zhang *et al* 2017b, Chen *et al* 2019, Chung *et al* 2019, Huang *et al* 2021, Han *et al* 2022, Msayib *et al* 2022, Wu *et al* 2022a, 2022b, Zhou *et al* 2023, Zhao *et al* 2023b). In contrast, the NOE(−1.6) is a relatively new signal whose dip can only be observed at high magnetic fields (>7 T). This NOE(−1.6) signal arises from dipolar interactions between the protons of the choline head group in phospholipids and water protons (Zu *et al* 2020). Hence, this signal holds potential as a novel molecular imaging mechanism for detecting choline phospholipids and their metabolism, which play an essential role in various pathologies and disorders (Zeisel and Canty 1993, Bodennec *et al* 2002, Glunde *et al* 2004, Hsu and Sabatini 2008, Hanahan and Weinberg 2011, Whiley *et al* 2014, Mori *et al* 2016, Javaid *et al* 2021). Prior research has demonstrated the potential of the NOE(−1.6) signal in diagnosing tumors and ischemic strokes in rodents at 9.4 T and 7 T, respectively (Zhang *et al* 2016, 2017a, Heo *et al* 2016a, Tee *et al* 2017). The signal has also been detected in the human brain at 9.4 T and in human blood at 7 T (Shah *et al* 2018). Moreover, it has demonstrated sensitivity to injury-associated apoptosis in mice kidneys with unilateral ureter obstruction (Wang *et al* 2017a) and injured spinal cords in monkeys (Wang *et al* 2021) at 7 T and 9.4 T, respectively.

Recently, we have validated the presence of the NOE(−1.6) effect in rat brains at a relatively lower magnetic field of 4.7 T (Viswanathan *et al* 2024b). However, upon directly observing the Z-spectra obtained with the commonly used saturation RF amplitudes (e.g. 0.5–1 *μ*T) at 4.7 T, we did not find a distinct NOE(−1.6) dip. This raises concerns about the accuracy and robustness of quantifying the NOE(−1.6) signal. Conventionally, an asymmetry analysis method has been employed to quantify the CEST effect by effectively reducing the confounding direct water saturation (DS) and semi-solid magnetization transfer (MT) effects (Guivel-Scharen *et al* 1998, Zhou *et al* 2003, 2011, 2022). However, this method tends to combine the contributions from CEST/NOE effects on both sides of the water line (Sun *et al* 2024). To address this issue, the multiple-pool model Lorentzian fit method has been commonly applied to separate a target CEST/NOE effect from the confounding factors (Zaiss *et al* 2011, Desmond *et al* 2014, Zhang *et al* 2017b). Nonetheless, the multiple-pool model Lorentzian fit method may struggle to robustly fit CEST/NOE effects close to the water resonance, where distinct dips are lacking. This issue becomes more significant under conditions of low magnetic fields (closer dips), relatively high saturation RF amplitudes (broader dips), or low signal-to-noise ratio (SNR). Additionally, its signal amplitude is reduced by the ‘shine through’ effect from the significant DS effect at low fields (Zaiss *et al* 2015b), leading to low SNR. As such, the multiple-pool model Lorentzian fit may not be suitable for quantifying the NOE(−1.6) signal at low magnetic fields. Therefore, exploring new methods to enhance the accuracy and robustness of quantifying the NOE(−1.6) signal, as well as enhancing SNR, is necessary for its low-field applications.

Machine learning (ML) models are capable of modeling complex, non-linear relationships and are not limited by a specific data distribution or shape. They can also be trained to deal with noisy data and outliers. Recently, ML methods have emerged as promising tools for quantifying CEST effects (Glang *et al* 2020, Kim *et al* 2020, Huang *et al* 2022), demonstrating superior robustness compared to the multiple-pool model Lorentzian fit. These ML models were trained on either *in vivo* data from measurements (Zaiss *et al* 2019, Glang *et al* 2020, Huang *et al* 2022, Liu *et al* 2022, Zhao *et al* 2022, Hunger *et al* 2023, Mohammed Ali *et al* 2023, Weigand-Whittier *et al* 2023, Yuan *et al* 2023) or fully simulated data from Bloch simulations (Chen *et al* 2020, Chen *et al* 2024, Kim *et al* 2020, Kang *et al* 2021, Bie *et al* 2022, Karunanithy *et al* 2022, Perlman *et al* 2022, 2023, Cohen *et al* 2023, Mohammed Ali *et al* 2023, Singh *et al* 2023, Yang *et al* 2023, Heo *et al* 2024). However, ML methods trained on measured data often face challenges due to limited data availability and a shortage of high-quality ground truth data. Currently, the CEST effects fitted by the multiple-pool model Lorentzian fit are being used as ground truth for ML training, which is not suitable for the NOE(−1.6). Additionally, when using fully simulated data, it is difficult to configure models and sample parameter ranges that closely resemble the measured data.

Most recently, we have developed a more practical and accurate ML method using a new type of partial synthetic data for training (Viswanathan *et al* 2024c). Unlike the traditional concept of partially synthetic data, this partially synthetic CEST data is not merely a mix of measured and simulated CEST signals. Instead, it reconstructs CEST signals by integrating all underlying components, including CEST, NOE, MT, and DS, derived from either measurements (i.e. measured components) or simulations (i.e. simulated components), using an inverse summation relationship (Zaiss *et al* 2015b). This method relies on measurements of a limited number of *in vivo* samples to ensure the accuracy of the data while using simulations to generate a large dataset that encompasses all possible variations in various pathologies. This approach effectively tackles the previous challenges. In this paper, we applied the ML method trained on this partial synthetic data to address the challenge of low accuracy and robustness in quantifying the NOE(−1.6) effect in an animal tumor model at 4.7 T.

## 2. Materials and methods

### 2.1. Animal preparation

This study involved seven rats, each bearing a 9 l tumor in the right hemisphere of the brain. The rats were anesthetized with 2% isoflurane and given oxygen during the MRI experiments. To ensure that their temperature remained at 37 °C, a warm-air feedback system (SA Instruments, Stony Brook, NY) was used. All animal procedures were approved by the Animal Care and Usage Committee of Vanderbilt University Medical Center.

### 2.2. MRI

CEST Z-spectra were acquired from rat brains by applying frequency offsets (Δ*ω*) ranging from −10 ppm to 10 ppm, totaling 89 data points. The frequency steps were set at 0.125 ppm intervals between −5 ppm and 5 ppm and 1.25 ppm intervals for other frequency ranges. Control images were acquired without applying the RF saturation. The CEST sequence consisted of a 5 s continuous wave (CW) rectangular saturation pulse, followed by a single-shot spin-echo echo planar imaging acquisition and a 2 s recovery period before the next saturation pulse. The saturation RF amplitude (*B*_1_ = *ω*_1_/*γ*, where *γ* is the gyromagnetic ratio of the proton) used was 1 *μ*T. The observed water longitudinal relaxation time (*T*_1obs_ = 1/*R*_1obs_) and the semi-solid MT pool size ratio (*f*_m_) were obtained using a selective inversion recovery quantitative MT method (Gochberg and Gore 2007). The image matrix size was 64 × 64, the field of view was 30 × 30 mm^2^, and the number of averages was 1. All measurements were carried out on a Varian 4.7 T magnet with a 38 mm receive coil at Vanderbilt University Institute of Imaging Science.

### 2.3. Multiple-pool model Lorentzian fit

The multiple-pool model Lorentzian fit was utilized to obtain the measured components for reconstructing the partially synthetic CEST signals, as well as to quantify the NOE(−1.6) effect for comparison with the predicted NOE(−1.6) effect using the ML model trained on the partially synthetic CEST signals. This Lorentzian model comprises six pools: amide, amine/guanidine (guan), water, NOE(−1.6), NOE(−3.5), and semi-solid MT. Supporting information table S1 lists the starting points and fit boundaries of the multiple-pool model Lorentzian fit. To quantify a specific CEST/NOE effect, a reference signal (*S*_ref_) was calculated by summing all Lorentzian fits, excluding the fit for the specific pool being analyzed. Meanwhile, a label signal (*S*_lab_) was derived by summing all the Lorentzian fits (except where it was noted). All fitted CEST/NOE spectra were calculated using an apparent exchange-dependent relaxation (AREX) metric (Zaiss *et al* 2015b), which inversely subtracts *S*_lab_ from *S*_ref_ along with *T*_1obs_ normalization. The amplitude (*A*) of the fitted NOE(−1.6) was determined by selecting the maximum values between −1 ppm and −2 ppm on the fitted NOE(−1.6) spectrum. The width (*W*) of the fitted NOE(−1.6) was determined by using the full width at half maximum of the fitted NOE(−1.6) spectrum within the same −1 ppm to −2 ppm range. Considering that the central frequency offset (Δ) of the fitted NOE(−1.6) peak varies around −1.6 ppm (Zhang *et al* 2017b), we used the fitted NOE(−1.6) amplitude and width to reconstruct the NOE(−1.6) peak using $A/\left( {1 + {{\left( {\Delta \omega - \Delta } \right)}^2}/{{\left( {0.5W} \right)}^2}} \right)$, where Δ is fixed at −1.6 ppm. The regenerated NOE(−1.6) peak provides a fair comparison with those from all ML predictions. In this paper, the multiple-pool model Lorentzian fitted NOE(−1.6) peak refers to this regenerated NOE(−1.6) peak.

### 2.4. Generation of partially synthetic CEST data

The formula to generate the partially synthetic CEST Z-spectrum for training ML models for predicting the NOE(−1.6) effect is provided in equation (1) (Viswanathan *et al* 2024c),

\begin{align*}\frac{{S\left( {\Delta \omega } \right)}}{{{S_0}}} &amp; = \frac{{{R_{1{\text{obs}}}}}}{{{R_{{\text{eff}}}}\left( {\Delta \omega } \right) + \frac{{R_{{\text{ex}}}^{{\text{NOE}}\left( { - 1.6} \right)}\left( {\Delta \omega } \right)}}{{1 + {r_{{\text{MT}}}}{f_{\text{m}}}}} + {r_{{\text{NOE}}\left( { - 3.5} \right)}}\frac{{R_{{\text{ex}}}^{{\text{NOE}}\left( { - {\text{3}}{\text{.5}}} \right)}\left( {\Delta \omega } \right)}}{{1 + {r_{{\text{MT}}}}{f_{\text{m}}}}} + {r_{{\text{amine/guan}}}}\frac{{R_{{\text{ex}}}^{{\text{amine/guan}}}\left( {\Delta \omega } \right)}}{{1 + {r_{{\text{MT}}}}{f_{\text{m}}}}} + {r_{{\text{MT}}}}R_{{\text{ex}}}^{{\text{MT}}}\left( {\Delta \omega } \right)}} \nonumber\\ &amp; \quad \cdot\frac{{\Delta {\omega ^2}}}{{\omega _1^2 + \Delta {\omega ^2}}}\end{align*}

where ${R_{{\text{eff}}}}$, $R_{{\text{ex}}}^{{\text{NOE}}\left( { - 1.6} \right)}$, $R_{{\text{ex}}}^{{\text{NOE}}\left( { - 3.5} \right)}$, $R_{{\text{ex}}}^{{\text{amine/guan}}}$, and $R_{{\text{ex}}}^{{\text{MT}}}$ represent the effective water relaxation, NOE(−1.6), NOE(−3.5), amine/guan CEST, and semi-solid MT effects in the rotating frame, respectively; ${r_{{\text{NOE}}\left( { - 3.5} \right)}}$, ${r_{{\text{amine/guan}}}}$, and ${r_{{\text{MT}}}}$ represent three scaling factors to adjust the amplitude of NOE(−3.5), amine/guan CEST, and semi-solid MT effect, respectively.

$R_{{\text{ex}}}^{{\text{NOE}}\left( { - 1.6} \right)}$ was a simulated component derived by evaluating a ${R_{{\text{ex}}}}$ formula in the following equation (2), using the concentration (*f*_s_), exchange/coupling rate (*k*_sw_), and solute transverse relaxation rate (*R*_2s_) for the corresponding pool, as well as the applied *ω*_1_ (Zaiss and Bachert 2013),

\begin{equation*}{R_{{\text{ex}}}}\left( {\Delta \omega } \right) = { }\frac{{{f_{\text{s}}}{k_{{\text{sw}}}}\omega _1^2}}{{\omega _1^2 + \left( {{R_{2{\text{s}}}} + {k_{{\text{sw}}}}} \right){k_{{\text{sw}}}} + \frac{{{{\left( {\Delta \omega - \Delta } \right)}^2}{k_{{\text{sw}}}}}}{{{R_{2{\text{s}}}} + {k_{{\text{sw}}}}}}}}.\end{equation*}

${R_{{\text{eff}}}}$ was another simulated component derived by evaluating the following equation,

\begin{equation*}{R_{{\text{eff}}}} = {R_{1{\text{obs}}}}\frac{{\Delta {\omega ^2}}}{{\omega _1^2 + \Delta {\omega ^2}}} + {R_{2{\text{w}}}}\frac{{\omega _1^2}}{{\omega _1^2 + \Delta {\omega ^2}}}.\end{equation*}

$R_{{\text{ex}}}^{{\text{NOE}}\left( { - 3.5} \right)}$, $R_{{\text{ex}}}^{{\text{amine/guan}}}$, and $R_{{\text{ex}}}^{{\text{MT}}}$ were measured components obtained from the multiple-pool model Lorentzian fit of the measured Z-spectra. $R_{{\text{ex}}}^{{\text{NOE}}\left( { - {\text{3}}{\text{.5}}} \right)}$ and $R_{{\text{ex}}}^{{\text{amine/guan}}}$ were obtained using the AREX metric and the multiple-pool model Lorentzian fitted *S*_lab_ and *S*_ref_ (Zaiss *et al* 2015b). Notably, to improve the robustness in the multiple-pool model Lorentzian fit, the amine and guanidine pools, which partially overlap, were treated as a single pool. Consequently, *S*_lab_, from the summation of all Lorentzian fits, cannot accurately represent these two CEST effects. Therefore, to produce a more accurate representation of the amine/guan CEST effect, we used the Z-spectrum as *S*_lab_ for quantifying $R_{{\text{ex}}}^{{\text{amine/guan}}}$ effect. $R_{{\text{ex}}}^{{\text{MT}}}$ was calculated using *R*_1obs_*L*_MT_/(1 − *L*_MT_) (Cui *et al* 2023a), where *L*_MT_ is the multiple-pool model Lorentzian fitted MT spectrum, derived from the subtraction of *S*_lab_ from *S*_ref_ for the MT pool. The APT effect was not considered in generating the partially synthetic CEST Z-spectra here, since it is far from the NOE(−1.6) effect and has negligible influence on the quantification of NOE(−1.6). The feasibility of using equation (1) for generating accurate partially synthetic CEST signals was validated by comparing the measured CEST signals with the reconstructed CEST signals using all components from the measurement fitting in supporting information figures S1 and S2. To generate *B*_0_ shift (Δ*ω*_shift_), Δ*ω* in equations (1)–(3) was replaced by Δ*ω* + Δ*ω*_shift_. Furthermore, the measured components were first interpolated, and Δ*ω* for each measured component was replaced by Δ*ω* + Δ*ω*_shift_. It is important to note that the Z-spectra used to derive these measured components should be corrected for *B*_0_ shifts beforehand.

A wide range of training data was created using equation (1) through the systematic adjustment of sample parameters such as *f*_s_, *k*_sw_, and longitudinal and transverse relaxation time (*T*_1_, *T*_2_) for the simulated components and *r* for the measured components, as well as *B*_0_ shift, as detailed in the supporting information table S2. The Z-spectrum, from which the measured components were derived, could be obtained either from an average of voxels in a region of interest (ROI) or from a single voxel, provided the SNR was sufficient. Multiple ROIs or voxels from various pathologies could be chosen to yield adequate features to encapsulate the variability of a particular disease. Partially synthetic data encompassing a broad spectrum of pathological conditions could be generated by utilizing multiple sets of measured components from these ROIs or voxels along with the simulations. The target NOE(−1.6) spectrum for this partially synthetic dataset was determined by calculating the corresponding ${R_{{\text{ex}}}}$ formula in equation (2) without setting the *B*_0_ shift. The target NOE(−1.6) peak amplitude (*A*) was calculated by setting Δ*ω* to Δ (i.e. −1.6 ppm) in equation (2), while the target NOE(−1.6) peak width (*W*) was determined by equation (4) (Zaiss *et al* 2011),

\begin{equation*}W \approx { }2\sqrt {\omega _1^2\frac{{{R_{2{\text{s}}}} + {k_{{\text{sw}}}}}}{{{k_{{\text{sw}}}}}} + {{\left( {{R_{2{\text{s}}}} + {k_{{\text{sw}}}}} \right)}^2}} .\end{equation*}

### 2.5. Measured CEST data and generation of fully simulated CEST data

The measured CEST Z-spectra on rat brains served as the data type for the ML method trained on measured data, with the multiple-pool model Lorentzian fitted NOE(−1.6) amplitude and width as training targets. The simulated CEST Z-spectra, created through the numerical simulation of a multiple-pool model Bloch–McConnell equation, served as the data type for the ML method trained on fully simulated data. A seven-pool model, consisting of the amide at 3.5 ppm, amine at 3 ppm, guanidine at 2 ppm, water, NOE(−1.6), NOE(−3.5), and semi-solid MT, as well as the same sequence parameters as in the measurements was used in this simulation. For a fair comparison of the ML methods trained on partially synthetic data and the fully simulated data, the sample parameters of water and NOE(−1.6) pool for this fully simulated data were identical to those for partially synthetic data. Details for other sample parameters and the *B*_0_ shift can be found in the supporting information table S3. The ground truth NOE(−1.6) spectrum for the fully simulated data was determined using equation (2) without setting the *B*_0_ shift. The ground truth NOE(−1.6) peak amplitude and width were obtained using the same methods as in obtaining the target NOE(−1.6) peak amplitude and width in partially synthetic data. This ground truth information will serve as the training targets in the ML training for fully simulated data.

### 2.6. ML training

**Figure 1.**
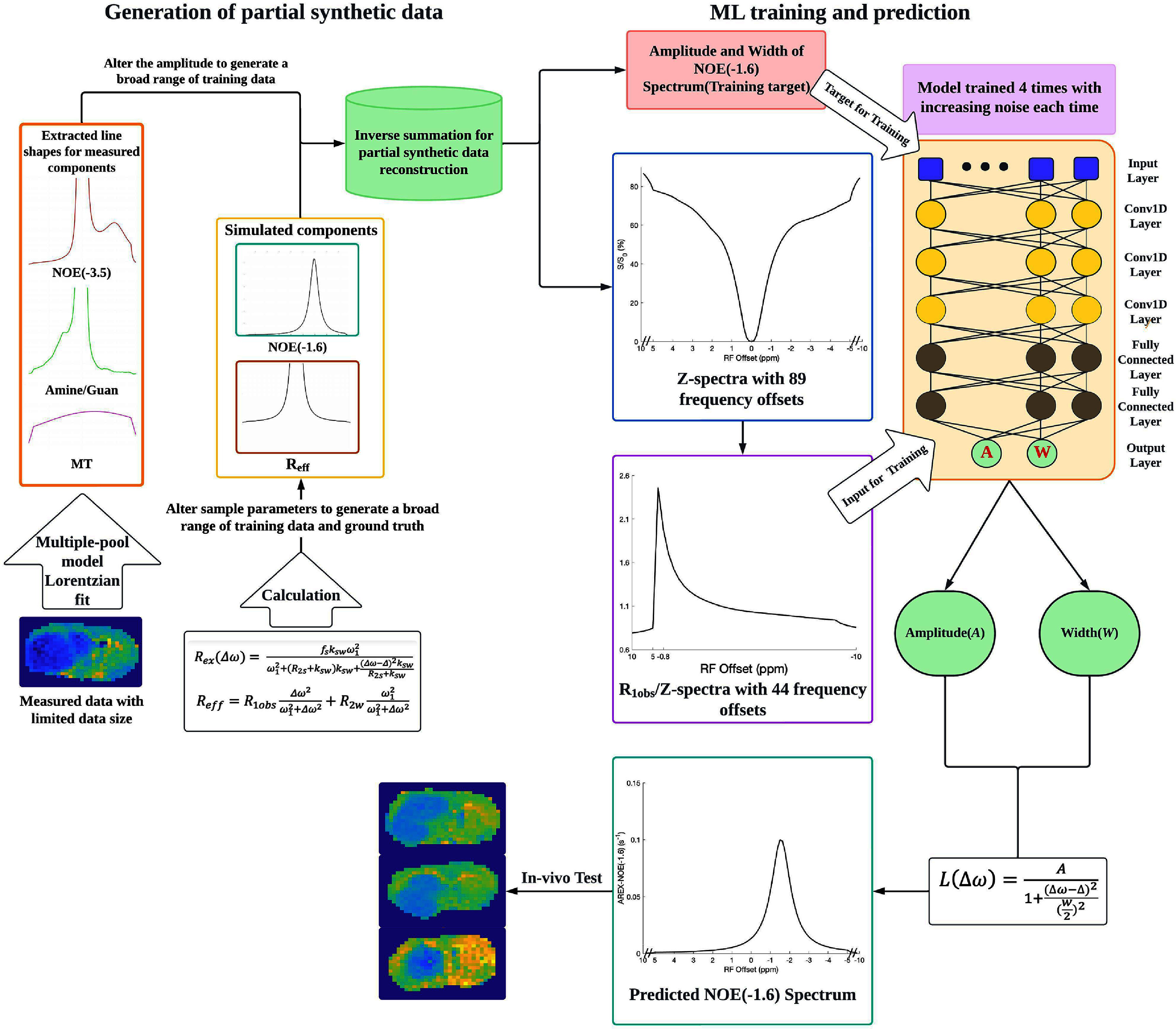
The flowchart for generating the partially synthetic CEST data as well as the NN model to predict the NOE(−1.6) effect for all data types.

Figure 1 shows a flowchart for generating the partially synthetic CEST data and the deep neural network (NN) to predict the NOE(−1.6) effect for all data types.

The input data contained *R*_1obs_/Z-spectrum with Δ*ω* spanning from −10 ppm to −0.8 ppm, and 5 ppm to 10 ppm at 4.7 T, totaling 44 data points. The output dataset included the amplitude (*A*) and width (*W*) of the NOE(−1.6) peak, which were separately trained. The predicted NOE(−1.6) spectrum was generated by putting the predicted *A* and *W* into $A/\left( {1 + {{\left( - \right)}^2}/{{\left( {0.5W} \right)}^2}} \right)$, where Δ is fixed at −1.6 ppm. The NN model incorporated three 1D convolutional layers with decreasing filter counts, a dropout layer, two dense layers with 256 and 128 neurons, respectively, and an output layer to predict *A* and *W* values. Each convolutional layer used the exponential linear unit activation function, and the training process utilized the mean square error loss function. Curriculum learning was implemented during the training to denoise CEST signals. This method involved repeating the training process four times and incrementally introducing Gaussian noise with a standard deviation of 0.005 to the original training data. The curriculum learning approach was only applied to training on partially synthetic data and fully simulated data. It was not used for measured data because of the unavailability of clean, noise-free datasets.

Since the ${R_{{\text{ex}}}}$ values can be obtained using the AREX metric, the predicted NOE(−1.6) effect was termed AREX-NOE(−1.6) in order to differentiate it from that using the conventional quantification metric which directly subtracts *S*_lab_ from *S*_ref_ (Zhou and van Zijl 2006, Cui *et al* 2023b). The NN model was implemented in MATLAB R2023b, using the Adam optimizer for 1000 epochs in the initial iteration and 100 epochs in subsequent iterations. The learning rate was set at 1 × 10^−3^ with a batch size of 32. All procedures, including the generation of all types of data as well as the training and testing pipelines, were executed on a MacBook Pro with the Apple M1 Chip, 8-core CPU, and 3.2 GHz processor. The generation of partially synthetic data was relatively quick, taking about 1–2 s. In contrast, generating fully simulated data of the same size was significantly more time-consuming, requiring approximately 10 h. Training with partially synthetic and fully simulated data took 15–20 min, while training with measured data, excluding augmentation datasets, took less than 10 s.

The training and validation datasets for all training tasks included in this work were partitioned to enable the model to assess its ability to fit the data across middle-level and side-level parameters effectively. Validation data was sampled from two non-contiguous ranges, spanning 5%–10% and 70%–80% of the dataset, ensuring coverage of diverse parameter conditions while maintaining separation from the training data. The remaining data was used for training, providing the model with a broad representation of the dataset. This partitioning strategy allows for a robust evaluation of the model’s generalization and fitting performance across varying parameter levels.

### 2.7. Generation of tissue-mimicking CEST data for validating the accuracy of the ML method trained on various types of data and the Lorentzian fit method

Tissue-mimicking CEST data, providing the ground truth, were utilized to evaluate the accuracy of both the ML method trained on various types of data and the multiple-pool model Lorentzian fit method in quantifying the NOE(−1.6) effect (Viswanathan *et al* 2024c). The tissue-mimicking data were generated through numerical simulations of the multiple-pool model Bloch–McConnell equations. Detailed sample parameters and the *B*_0_ shift used to generate this dataset are provided in supporting information table S4. The ground truth NOE(−1.6) spectrum for these data was obtained by calculating the corresponding ${R_{{\text{ex}}}}$ formula in equation (2) without setting a *B*_0_ shift. We generated 177 147 tissue-mimicking Z-spectra and selected 1000 of them, along with their ground truth values, as the testing dataset. To evaluate the effectiveness of different types of training data in predicting the NOE(−1.6) effect on tissue-mimicking data, we created several datasets using methods similar to those used in our following *in vivo* evaluation. These datasets include: partially synthetic data, measured data, measured data with augmentation, fully simulated data, and both fully simulated and measured data. To emulate partially synthetic data for tissue-mimicking validation, we applied the multiple-pool model Lorentzian fit to one or more Z-spectra from the remaining tissue-mimicking data to extract the measured components. The average of these fitted components was then used to generate the partially synthetic data. The ML model trained on this dataset was subsequently used to process other Z-spectra in the tissue-mimicking dataset to predict the NOE(−1.6) effect. To emulate measured data for tissue-mimicking validation, we randomly selected another 1000 tissue-mimicking Z-spectra from the remaining tissue-mimicking data and used their Lorentzian-fitted results as training targets. To emulate measured data with augmentation for tissue-mimicking validation, we enlarged the measured dataset by averaging every pair of Z-spectra. The training targets were derived from the Lorentzian fits of these augmented inputs. To emulate fully simulated data for tissue-mimicking validation, we employed the simulation of a multiple-pool model Bloch–McConnell equation as previously described, using nearly identical sample parameters detailed in supporting information table S3. Since the fully simulated data and the tissue-mimicking data were generated using similar parameters and the same simulation model, it was essential to introduce variability to distinguish between them and to mimic the inherent differences between fully simulated data and *in vivo* CEST signals. Each parameter unrelated to NOE(−1.6) used to generate the fully simulated data was randomly assigned a multiplier of 1 ± 0.3 for generating each Z-spectrum. For instance, the same parameter could be multiplied by 0.7 for one Z-spectrum and by 1.3 for another. To emulate both fully simulated and measured data for tissue-mimicking validation, the ML model was first trained on fully simulated data and then fine-tuned on measured data for an additional 1000 epochs. Supporting information table S5 lists the sample sizes for all types of data used in this validation. The ML models trained on these various data types were then applied to predict the NOE(−1.6) effect on the testing tissue-mimicking data. The absolute difference between the predicted NOE(−1.6) amplitude and the ground truth NOE(−1.6) amplitude, referred to as loss, was calculated as a measure of the ML model’s accuracy. In addition, the absolute difference between the multiple-pool model Lorentzian fitted NOE(−1.6) amplitude and the ground truth NOE(−1.6) amplitude was also obtained.

### 2.8. Comparison of the performance of various ML methods trained on different types of data with the Lorentzian fit on *in vivo* data

To assess the effectiveness of the ML method trained on partial synthetic CEST data compared to other data types for *in vivo* prediction, we conducted two types of comparisons using data from different numbers of voxels and various brain regions (Viswanathan *et al* 2024c). For the first type of comparison, we first took an average of Z-spectra from various numbers of voxels (100, 500, 1000, and 1525), respectively, randomly selected within the measured results from four training rats. Notably, 1525 is the number of all voxels in the four training rats. Subsequently, the measured components were extracted from the multiple-pool model Lorentzian fit of these Z-spectra, and were employed to create partial synthetic data to train the ML model. Furthermore, the ML model was directly trained on all measured Z-spectra from each voxel that was used in the generation of partially synthetic data for the different numbers of voxels, respectively. The training data were also augmented by averaging every pair of Z-spectra from all voxels present in the four training rat brains (1525 voxels), which resulted in an increase of about 700 times in the original dataset size. The ML model was then applied to the measured Z-spectra from the remaining three testing rats. For the second type of comparison, we first obtained an average of Z-spectra from all voxels in the tumor regions (647 voxels) and the contralateral normal regions (547 voxels) of the seven rats. Subsequently, the multiple-pool model Lorentzian fit of these Z-spectra was performed to derive the measured components from either tumor regions or contralateral normal regions, which were then used to generate the partially synthetic data. Furthermore, we created another dataset including these two sets of partially synthetic data with measured components from both tumors and contralateral normal regions, doubling the data size compared to using components from either tumor or contralateral normal regions alone. The ML model was then trained on these partially synthetic data. In addition, the ML model was directly trained on all measured Z-spectra from each voxel in tumor regions, contralateral normal regions, and both these two regions (1194 voxels). The training data was also augmented by averaging each pair of Z-spectra from all voxels in the tumor and contralateral normal regions of the seven rats, expanding the dataset to nearly 300 times its original size. The model was then used to process the data from other parts of the seven rat brains. To evaluate the advantage of the ML method trained on the partially synthetic CEST data versus fully simulated data on animals, we also conducted two types of comparisons with different simulation models. These simulations involved varying the amine concentration in one type while it remained fixed at 0.3% in the other type. To evaluate the performance of ML training using both simulated and measured data, the ML model was initially trained on fully simulated data with varying amine concentrations and subsequently fine-tuned on measured data for an additional 1000 epochs. Supporting information table S6 details the data sizes for all types of training datasets used in the ML training for this comparative analysis.

### 2.9. Data analysis and statistics

All NOE(−1.6) maps display the distribution of amplitude derived from either the multiple-pool model Lorentzian fit or ML prediction. The tumor ROIs were delineated on the *T*_1obs_ maps, while the contralateral normal ROIs were selected to mirror the tumor ROIs. Student’s *t*-test was employed to evaluate the statistical difference between the tumors and contralateral normal tissues. A *p*-value less than 0.05 was considered to be statistically significant.

## 3. Results

**Figure 2.**
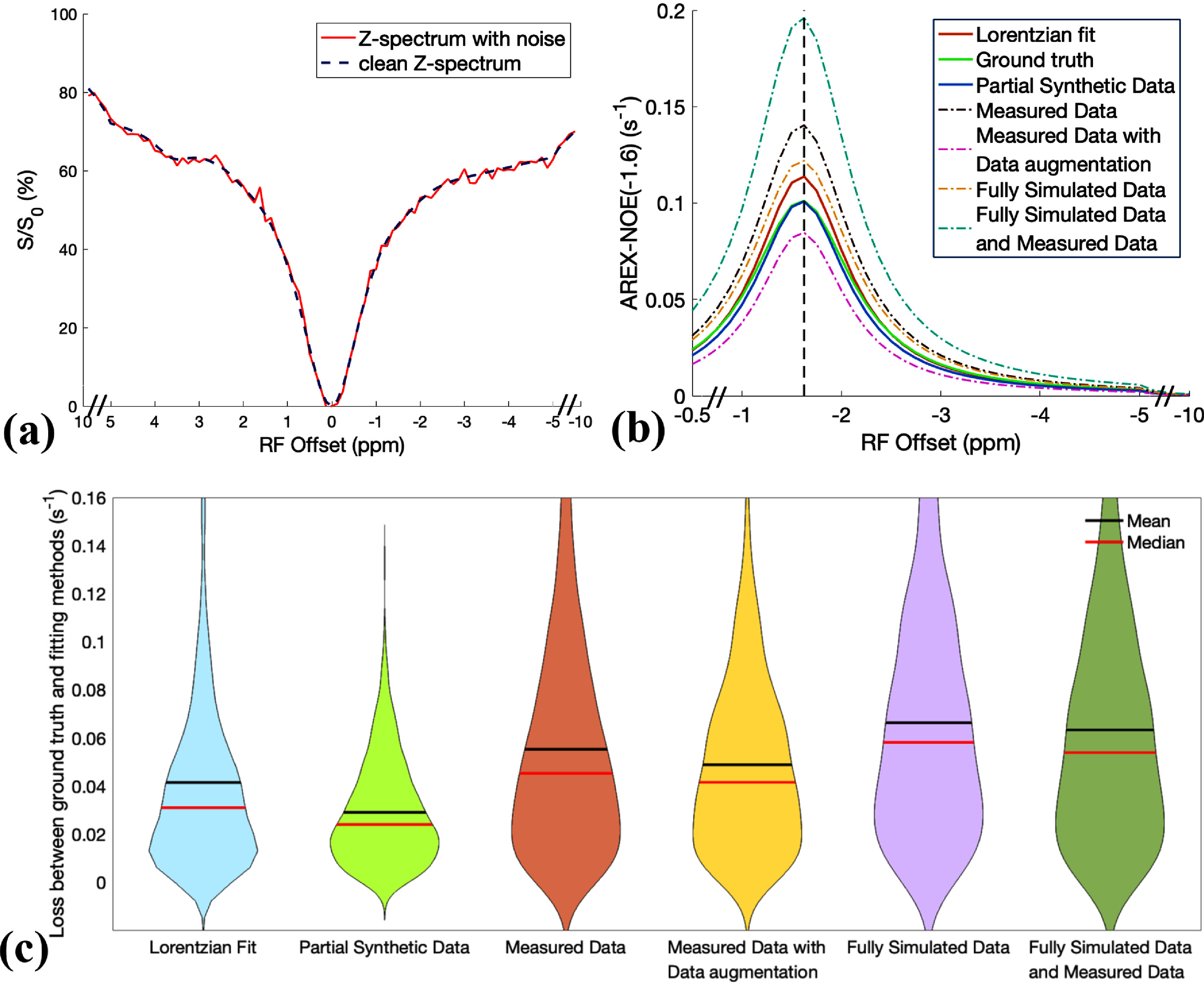
(a) A representative Z-spectrum from the tissue-mimicking data without (clean) and with added noise at an SNR of 75. (b) A comparison of the corresponding NOE(−1.6) spectra from the ML prediction using model trained on various types of data, multiple-pool model Lorentzian fit, and ground truth. Notably, the NOE(−1.6) spectrum from the ML model using partially synthetic data closely aligns with the ground truth. (c) A violin plot showing the losses between the ground truth and the multiple-pool model Lorentzian fit, and the losses between the ground truth and the ML prediction using model trained on various types of data, for all 1000 testing tissue-mimicking samples. The ML model was trained on partially synthetic data with the measured components fitted from a randomly selected Z-spectrum within the tissue-mimicking data.

Figure 2(a) exhibits a representative Z-spectrum from the tissue-mimicking data without/with added noise at an SNR of 75. Figure 2(b) presents a comparison of the corresponding NOE(−1.6) spectra from the ground truth data, multiple-pool model Lorentzian fit, and ML predictions. The ML model was trained on partially synthetic data with measured components from a randomly selected Z-spectrum within the tissue-mimicking data, as well as on various other types of data. Notably, the ML model trained on the partially synthetic data provides a closer approximation of the NOE(−1.6) spectrum to the ground truth compared to both the multiple-pool model Lorentzian fit and the ML models trained on other data types. Figure 2(c) shows the losses between the ground truth and the predicted results using these methods, for all testing tissue-mimicking samples with an SNR of 75 for the added noise. Specifically, the mean loss stands at 0.029 for the ML trained on the partially synthetic data, while 0.041 for the multiple-pool model Lorentzian fit method as well as 0.055 for measured data, 0.049 for measured data with augmentation, 0.066 for fully simulated data, and 0.063 for the combination of the fully simulated and measured data. Supporting information figure S3 shows the losses between the ground truth and the predicted results using the ML trained on partially synthetic data with measured components fitted from five other randomly selected Z-spectra separately, as well as from the average of 50, 500, and 5000 randomly selected Z-spectra within the tissue-mimicking data. Similar results were found in that the ML method has lower loss than all other methods. Supporting information figure S4 shows the results in figure 2(c) with various SNRs of the added noise. The mean loss for the ML method using partially synthetic data stands at 0.025, 0.027, and 0.030, while it is 0.031, 0.035, and 0.053 for the multiple-pool model Lorentzian fit method, for the added noise at SNR of 200, 100, and 50, respectively. Notably, the multiple-pool model Lorentzian fit is sensitive to noise, whereas our method is more robust to noise. Additionally, the ML model trained on partially synthetic data demonstrates the smallest loss compared to ML models trained on other data types across these noise levels. Collectively, these results confirm the superior accuracy and noise robustness of our ML method in predicting the NOE(−1.6) effect.

**Figure 3.**
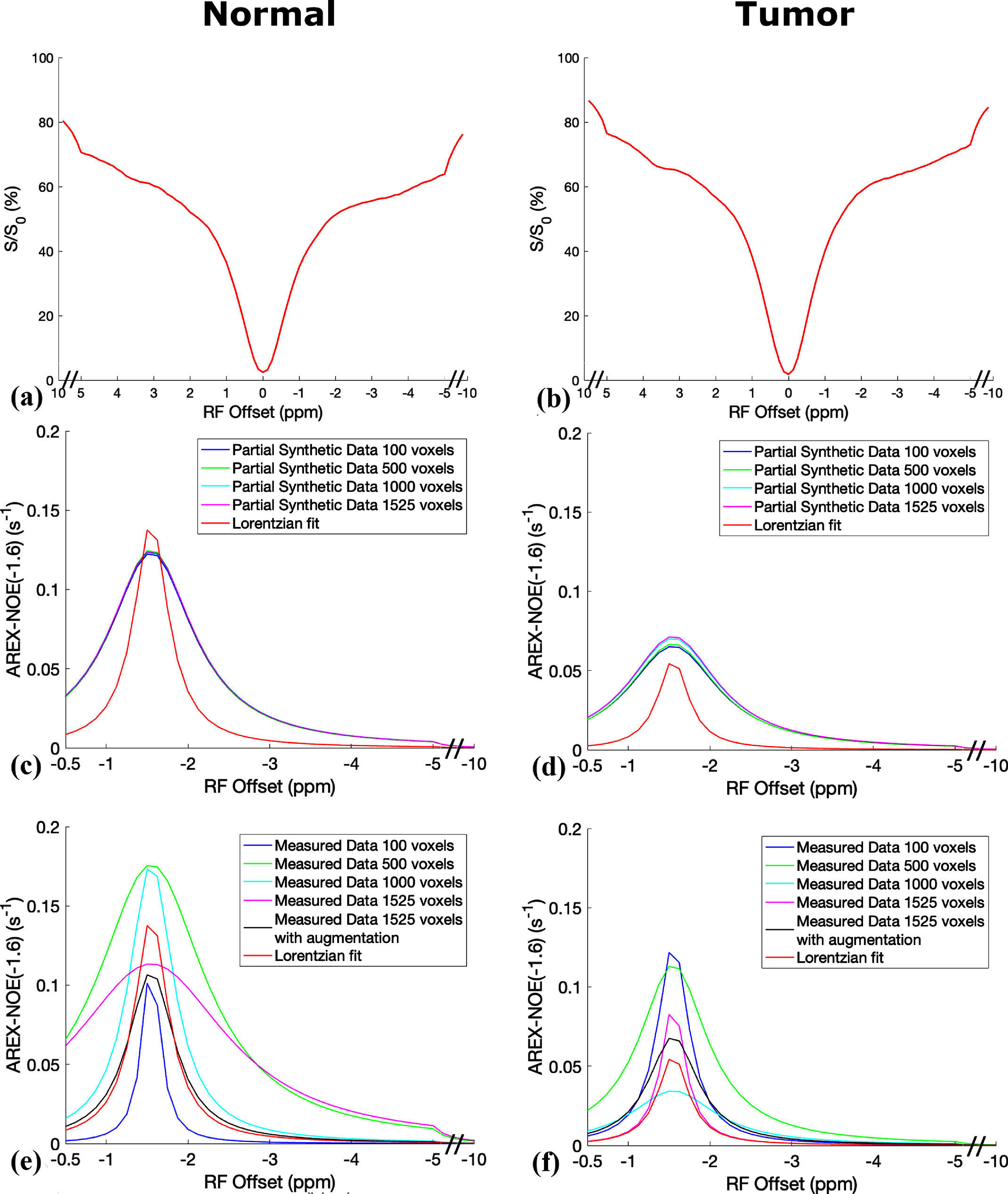
Average of the measured CEST Z-spectra from contralateral normal tissues and tumor tissues in the three testing rats for the first type of comparison (a), (b); the corresponding averaged NOE(−1.6) spectra from the ML prediction using partially synthetic data with measured components derived from the average of measured Z-spectra from 100, 500, 1000, and 1525 voxels, respectively, within the four training rat brains (c), (d); the corresponding averaged NOE(−1.6) spectra from the ML prediction using measured Z-spectra from 100, 500, 1000, and 1525 voxels, respectively, as well as from 1525 voxels with data augmentation within the four training rat brains (e), (f). The corresponding averaged NOE(−1.6) spectra from the multiple-pool model Lorentzian fit were also plotted in (c)–(f) for comparative analysis. The data are averaged across various subjects.

**Figure 4.**
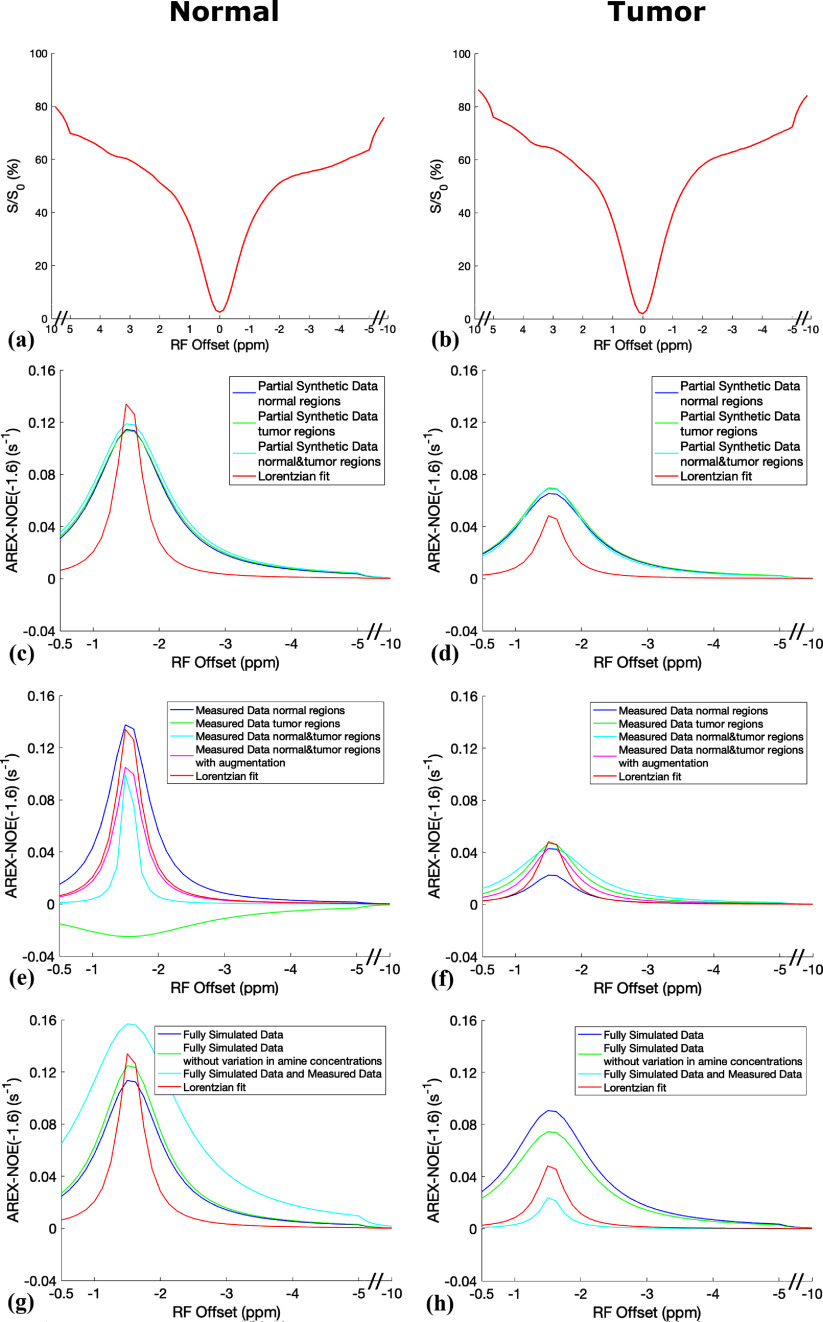
Average of the measured CEST Z-spectra from contralateral normal tissues and tumor tissues in the seven rats for the second type of comparison (a), (b); the corresponding averaged NOE(−1.6) spectra from the ML prediction using partially synthetic data with measured components derived from the average of measured Z-spectra from the contralateral normal regions, tumor regions, and both regions (c), (d); the corresponding averaged NOE(−1.6) spectra from the ML prediction using the measured Z-spectra from the contralateral normal regions, tumor regions, and both regions, as well as both regions with data augmentation (e), (f); the corresponding averaged NOE(−1.6) spectra from the ML prediction using fully simulated data with and without variation in the amine concentration, and using both fully simulated data and measured data (g), (h). The corresponding averaged NOE(−1.6) spectra from the multiple-pool model Lorentzian fit were also plotted in (c)–(h) for comparative analysis. The data are averaged across various subjects.

Figure 3 exhibits the average CEST Z-spectra from the contralateral normal tissue and tumors of the three testing rats for the first type of comparison, alongside the corresponding average NOE(−1.6) spectra derived from the multiple-pool model Lorentzian fit, ML prediction utilizing partially synthetic data and measured data from different numbers of voxels, as well as with data augmentation. Figure 4 presents the average CEST Z-spectra from the contralateral normal tissues and tumors of the seven rats for the second type of comparison. Additionally, it shows the corresponding average NOE(−1.6) spectra derived from the multiple-pool model Lorentzian fit, as well as ML predictions using models trained on various types of data including partially synthetic data, measured data from different brain regions, fully simulated data with different simulation models, and both fully simulated and measured data, supporting information figures S5 and S6 depict these plots in figures 3 and 4, respectively, inclusive of the standard deviation across subjects. The ML-predicted NOE(−1.6) spectra trained on all types of partially synthetic data exhibited a narrower range of variation compared to those trained on all types of measured data. This indicates that the partially synthetic approach provides greater robustness to variations in training data size, especially when the measured data is limited in quantity or lacks sufficient features. In contrast, the NOE(−1.6) spectra predicted by the ML model trained on fully simulated data using two different simulation types showed significant discrepancies, underscoring the heavy reliance of this approach on the specific simulation model. The ML prediction from model trained on both fully simulated and measured data also failed to accurately capture the patterns of the NOE(−1.6) effect.

**Figure 5.**
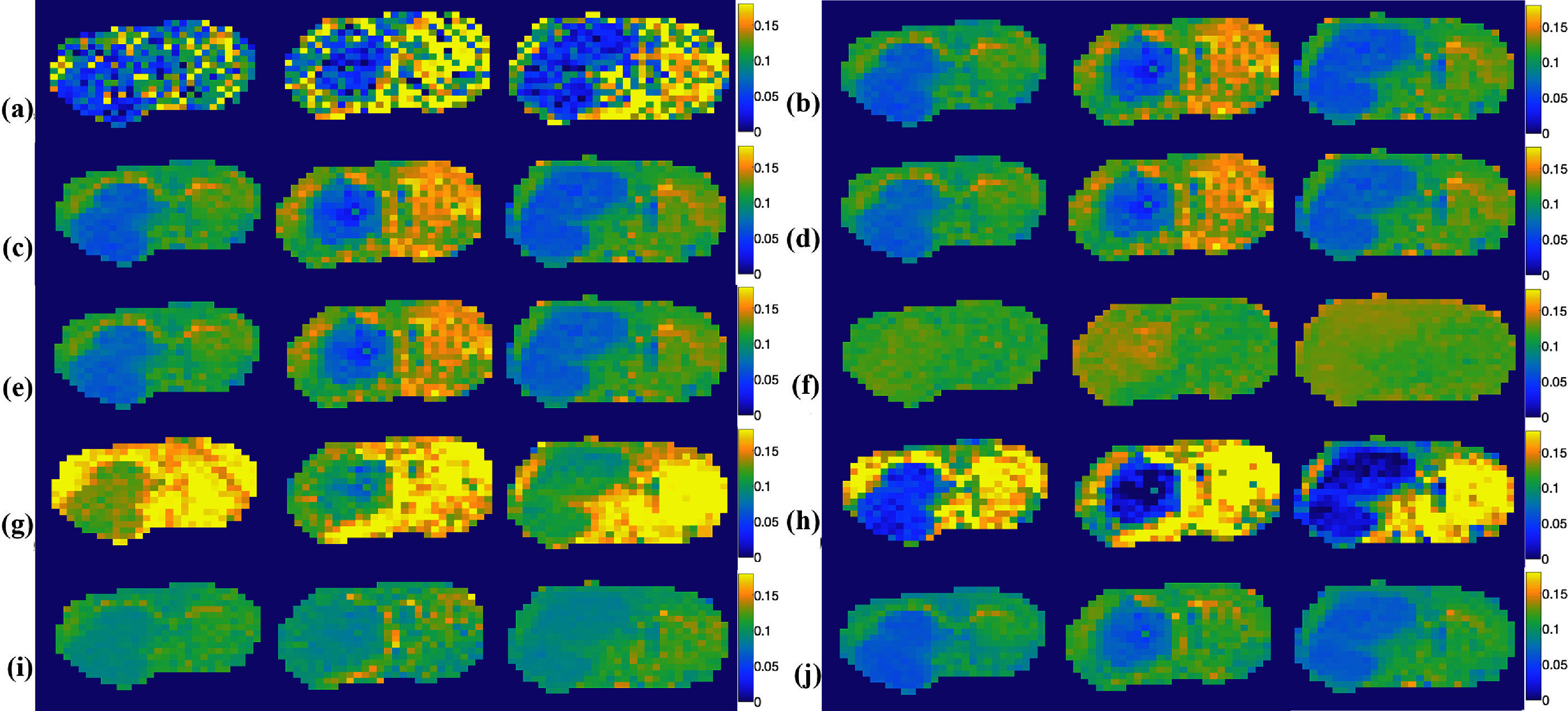
NOE(−1.6) amplitude maps from three testing rat brains for the first type of comparison using the multiple-pool model Lorentzian fit (a); ML prediction using partially synthetic data with the measured components from the average of measured Z-spectra from 100 (b), 500 (c), 100 (d), and 1525 (e) voxels, respectively, in the four training rat brains; ML prediction using the measured Z-spectra from 100 (f), 500 (g), 1000 (h), and 1525 (i) voxels, respectively, as well as from 1525 voxels with data augmentation in the four training rat brains (j).

**Figure 6.**
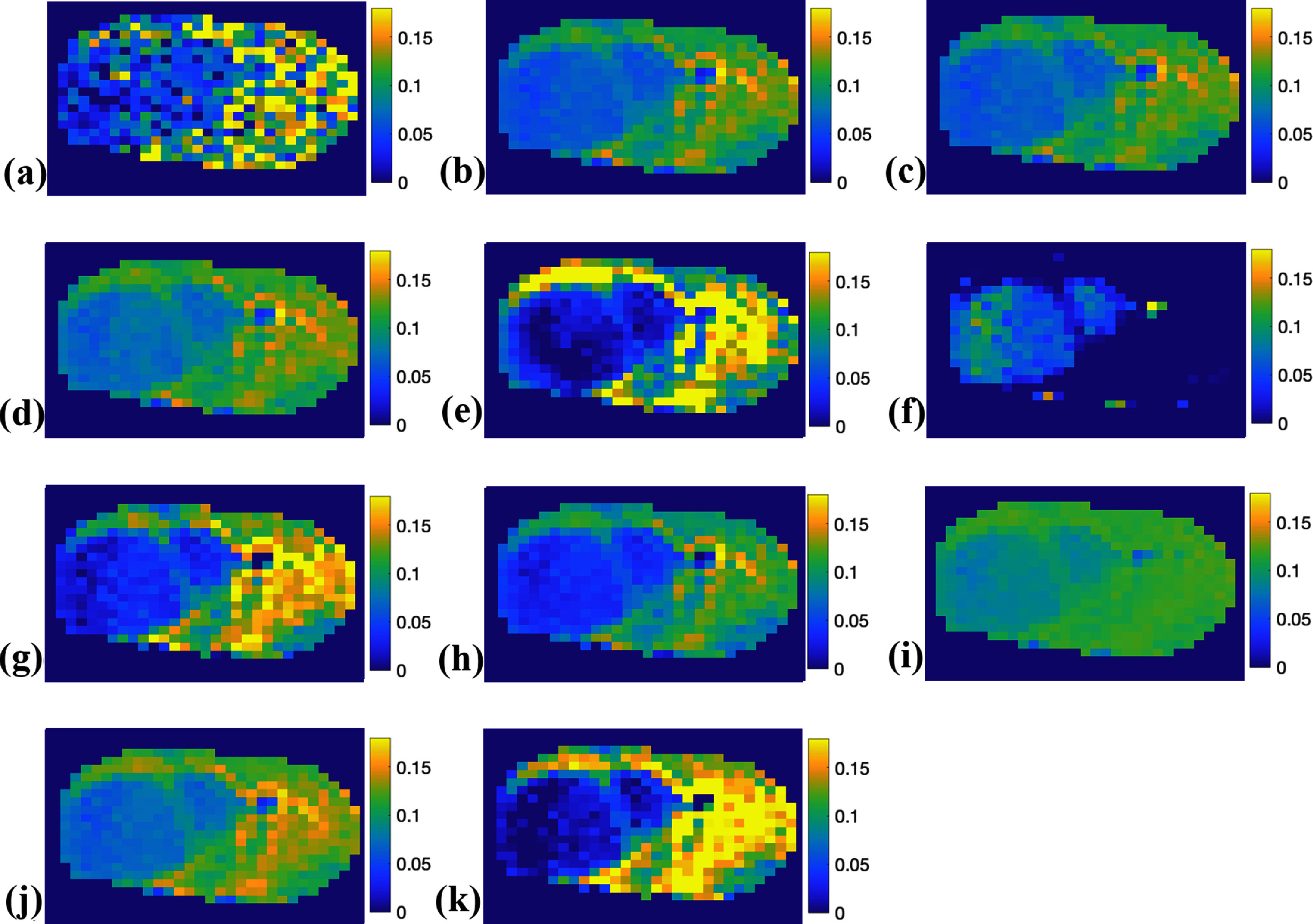
NOE(−1.6) amplitude maps from a representative rat brain for the second type of comparison using the multiple-pool model Lorentzian fit (a); ML prediction using partially synthetic data with measured components from the average of measured Z-spectra from the contralateral normal regions (b), tumor regions (c), as well as both regions (d); ML prediction using the measured Z-spectra from the contralateral normal regions (e), tumor regions (f), both regions (g), as well as both regions with data augmentation (h); ML prediction using fully simulated data with (i) and without (j) variation in the amine concentration; ML prediction using both fully simulated data and measured data (k).

Figure 5 presents the NOE(−1.6) amplitude maps from the brains of the three testing rats for the first type of comparison, obtained using the multiple-pool model Lorentzian fit and the ML prediction using partially synthetic data and measured data from various numbers of voxels and with data augmentation. Figure 6 illustrates the NOE(−1.6) amplitude maps from a representative rat brain for the second type of comparison, acquired using the multiple-pool model Lorentzian fit, ML prediction using partially synthetic data and measured data from different brain regions and with data augmentation, ML prediction using fully simulated data with different simulation models, and ML prediction using both fully simulated data and measured data. Supporting information figure S7 displays these NOE(−1.6) amplitude maps shown in figure 6 but derived from all seven rat brains. It was noted that all ML-predicted NOE(−1.6) amplitude maps have a higher SNR than those obtained through the multiple-pool model Lorentzian fit, indicating the superior denoising ability of ML methods. Moreover, the contrast between tumors and normal contralateral tissues using the ML model trained on partially synthetic data is more consistent compared to ML models trained on other data types.

**Figure 7.**
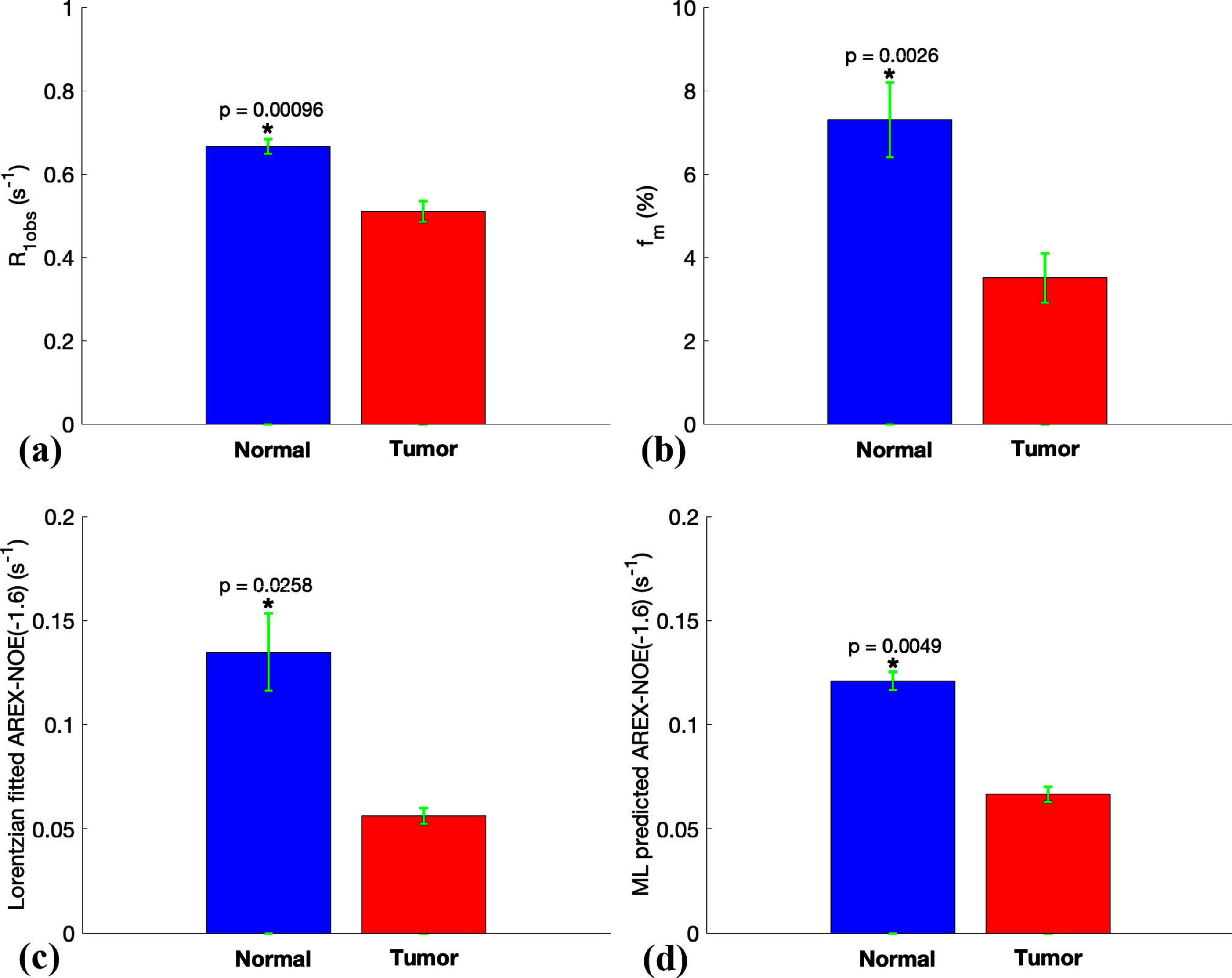
Statistical differences in *R*_1obs_ (a), *f*_m_ (b), the multiple-pool model Lorentzian fitted NOE(−1.6) amplitude (c), and the ML predicted NOE(−1.6) amplitude using partially synthetic data with measured components from the average of measured Z-spectra from 500 voxels in the four training rat brains for the first type of comparison (d), between the contralateral normal and tumor tissues in the three testing rat brains. (* *p* < 0.05).

Figure 7 presents the statistical difference in *R*_1obs_, *f*_m_, the multiple-pool model Lorentzian fitted NOE(−1.6) amplitude, and the ML predicted NOE(−1.6) amplitude obtained using the partially synthetic data with measured components from the average of 500 voxels in the four training rat brains for the first type of comparison. All these parameters show significant differences between the tumor and the contralateral normal tissues. In addition, tumors exhibit significantly lower NOE(−1.6) amplitudes compared to normal tissues, which aligns with previous findings (Zhang *et al* 2017a, Zu 2018, Viswanathan *et al* 2024b).

## 4. Discussion

The NOE(−1.6) is closer to the water peak compared to other CEST/NOE effects, falling around the shoulder of the Z-spectrum, especially at low magnetic fields. Consequently, it does not display a distinct dip, making it challenging to observe directly and complicating the ability of conventional quantification methods to isolate the NOE(−1.6) effect from others. ML methods have proven to be practical tools for identifying and quantifying features in complex data sets. They operate by recognizing patterns in the input data and using these patterns to make predictions, allowing them to identify complex features and patterns that would be challenging or impossible to detect using traditional methods. Our study suggests that ML models trained on partially synthetic data can significantly enhance the accuracy and robustness of predicting the NOE(−1.6) at the low field of 4.7 T. Specifically, our validation, which involved generating tissue-mimicking data with variations across all pools, demonstrates that our ML method can effectively minimize interference from other effects. Additionally, training ML models on partially synthetic data requires a much smaller data size from measurements than training them on measured data. Furthermore, it does not need to consider the complex model of exchangeable pools and also requires much less simulation time compared to those trained on fully simulated data, thus making it a more practical ML method.

The performance of the multiple-pool model Lorentzian fit method in quantifying the NOE(−1.6) effect was suboptimal, as shown by significant deviations from the ground truth and noisy fitted NOE(−1.6) maps in figures 3–6. This poor performance is likely due to the proximity of the NOE(−1.6) effect to the water peak, which causes overlap and interference. This interference complicates the method’s ability to accurately determine amplitudes within the specified frequency offset ranges, leading to distorted fitted curves and erroneous amplitude estimations. Supporting information figure S9 presents the original multiple-pool model Lorentzian fitted NOE(−1.6) spectra for random tissue-mimicking samples, which were not reconstructed by using their amplitudes and widths. These spectra exhibit significant distortions, indicating unreliable fitting. This also explains why the multiple-pool model Lorentzian fit amplitude maps in figures 5 and 6 exhibit a lower SNR and rougher texture. The amplitudes fitted by this method tend to be dominated by stronger and more distinct signals, leading to increased variability and irregularity in the amplitude maps.

For the ML model trained on measured data, we observed significant deviation in the predicted NOE(−1.6) from its training target (i.e. the multiple-pool model Lorentzian fitted NOE(−1.6)). This was noted even when the training data size reached 1162 300 and 358 312 for the different voxel number and brain region selections with data augmentation, respectively. This may be attributed to the fact that most of the data are derived from voxels with similar pathologies, which display similar patterns and, hence, do not provide diverse information. In contrast, each sample in the partially synthetic data contains different patterns, achieved by varying the combination of underlying sample parameters for the simulated components and the scaling factors for the measured components.

For the ML model trained on fully simulated data, we observed a significant influence from the variation of the amine CEST pool on the prediction. This could be due to the extension of the broad amine CEST peak to the upfield frequency range of the input data (i.e. beyond −0.8 ppm). As a result, when the amine CEST pool variation is absent in the training data, the ML model fails to learn the feature necessary to separate the NOE(−1.6) from the amine CEST effects, leading to inaccurate predictions. Notably, our previous study demonstrated that that the amine CEST effect cannot be ignored with the commonly used saturation RF fields in brains (Sun *et al* 2023). Potentially, including more simulation pools and/or using a broader range of sample parameters could encompass all possible variations. However, generating a larger dataset through numerical simulations of multiple-pool model Bloch equations requires more simulation time. An alternative approach has been used, which first fits some of the underlying sample parameters, including the pool concentration, exchange rate, and relaxations, and then generates the fully simulated data based on the fitted sample parameters (Bie *et al* 2022). This method is different from our concept of partially synthetic data, as it still relies on Bloch simulations which require the setting of all sample parameters. In contrast, our partially synthetic platform is based on the inverse summation relationship between the CEST signals and CEST/NOE effects from each pool. Importantly, fitting the sample parameters first requires the isolation of each CEST pool, which is not necessary if the major CEST effects can be fitted and used to reconstruct the training Z-spectra using our partially synthetic platform.

Figure 2(b) shows the multiple-pool model Lorentzian fitted NOE(−1.6) amplitude is higher than that obtained from the ML method when using partially synthetic data. However, this observation is based on a single sample of our tissue-mimicking data and does not establish a consistent pattern indicating whether Lorentzian fitted values are generally higher or lower than those obtained from our ML method on tissue-mimicking data. Additionally, this observation is not related to figure 3, which presents the average of the predictions and fitted values from all selected *in vivo* voxels. The variations in the Lorentzian fitted NOE(−1.6) amplitude on tissue-mimicking data arise from differences in sample parameters during the tissue-mimicking simulation. Supporting information figure S10 displays another version of figure 2, showing the original losses without taking their absolute values, highlighting both positive and negative deviations from the ground truth.

The use of ‘*R*_1obs_/Z-spectrum’ as input was inspired by the inverse summation relationship and the AREX metric (Zaiss *et al* 2015b). Since all CEST/NOE/MT/DS effects can be inversely summed to generate the Z-spectrum, using the inverse of the Z-spectrum as input can reduce the complexity of the data. In addition, CEST signals are influenced by *R*_1obs_. In order to specifically quantify the NOE(−1.6) effect, the *R*_1obs_ information needs to be included in the model to eliminate its contribution. The AREX metric suggests that *R*_1obs_ contribution can be removed by the formula ‘*R*_1obs_/Z-spectrum’; hence we chose to use this formula as an input. Furthermore, rather than utilizing the complete Z-spectrum as input, we excluded signals from −0.8 ppm to 5 ppm. This selection was based on the fact that the *R*_1obs_/Z-spectrum value at around 0 ppm is a singularity and many downfield CEST effects do not contribute to the upfield NOE effects, with the exception of the fast exchange amine CEST effect. To justify this input data formula and selection of RF frequency offset range, we obtained the losses between the ground truth and the ML method using partially synthetic data for a few different combination of input data formula (i.e. *R*_1obs_/Z-spectrum, Z-spectrum/*R*_1obs_, or concatenation of Z-spectrum and *R*_1obs_) and the selection of RF frequency offset range (i.e. part or complete Z-spectrum) in supporting information figure S8. We found that the mean losses for all these combinations were higher than that in our paper. Moreover, in this study, we used even data acquisition for the input data from −5 ppm to −0.8 ppm. Given that the NOE(−3.5) effect is broad, a more sparse acquisition should provide enough information to separate it from the NOE(−1.6). Future work could focus on optimizing the frequency ranges of the input data.

In this study, we chose to use a model with convolutional layers over others, such as the one with only fully connected layers, due to its effectiveness in processing sequential CEST signal data. Convolutional layers extract localized features, enhancing prediction accuracy and efficiency. Unlike models with only fully connected layers, which may miss important features, a convolutional model aligns weights with data labels during training, improving both robustness and accuracy (Remeseiro and Bolon-Canedo 2019, Pudjihartono *et al* 2022).

This study indicates potential for application in human imaging at low-field clinical scanners. However, to make this transition, several issues must be addressed. First, in human imaging, pulsed-CEST imaging sequence with a certain duty cycle (DC) is typically used. Fortunately, an analytical solution for the pulsed saturation has been derived using an approximating model of repeated hard pulses with a certain DC and the rectangular pulse amplitude, and the pulsed-CEST signal has been described using a modified inverse summation relationship (Gochberg *et al* 2018). This modified inverse summation relationship enables us to adapt the concept of the partially synthetic data from the CW saturation to pulsed saturation. Second, non-steady-state acquisition is commonly employed in human imaging. The non-steady-state partially synthetic data can be obtained using a previously developed equation to convert the steady-state CEST signal to a non-steady-state signal (Wang *et al* 2017b, Sun 2021, Xiao *et al* 2023, Zhou *et al* 2024). Third, the human brain has more complex structures. Including measured components from more regions such as grey matter, white matter, and cerebrospinal fluid could provide sufficient data features to enhance prediction accuracy. Finally, this study did not incorporate the *B*_1_ shift into the training data, as it is not severe in preclinical scanners. In human imaging, the *B*_1_ shift, defined as the ratio between the actual and the nominal RF saturation amplitude, can be simulated by multiplying it with *ω*_1_ in equations (2) and (3) for ${R_{{\text{eff}}}}$ and $R_{{\text{ex}}}^{{\text{NOE}}\left( { - 1.6} \right)}$, by multiplying it with $r$ in equation (1) for $R_{{\text{ex}}}^{{\text{NOE}}\left( { - 3.5} \right)}$, and by multiplying its square with $r$ in equation (1) for $R_{{\text{ex}}}^{{\text{amine/guan}}}$ and $R_{{\text{ex}}}^{{\text{MT}}}$ (Zhao *et al* 2023b, Viswanathan *et al* 2024a).

Based on our previous mechanism study (Zu *et al* 2020), a decrease in the NOE(−1.6) within a tumor may indicate changes in choline phospholipid properties in cell membrane. However, further validation is required to confirm this. Improving the methodology for more accurate and robust quantification of NOE(−1.6) effect as well as the underlying coupling rate using an ML approach could be beneficial for subsequent validation studies. Notably, figure 7 implies that the conventional multiple-pool model Lorentzian fit method might have overestimated the NOE(−1.6) contrast between tumor and normal tissues, as compared to the ML prediction.

## 5. Conclusion

The ML method trained on partially synthetic data can quantify the NOE(−1.6) effect at low fields with significantly improved accuracy and robustness compared to other existing methods.

## Data Availability

The data that support the findings of this study are openly available at the following URL/DOI: https://github.com/CESTlabZu/ML_NOE–1.6-.
